# Ameliorative effect and potential mechanism of hyperoside on diabetic retinopathy

**DOI:** 10.3389/fendo.2025.1556310

**Published:** 2025-05-02

**Authors:** Xu Yu, Hao Wu, Lei Zhou, Gaoxiang Wang, Xinyi Sun, Junjun Miao, Meijie Ben, Rongwei Shi, Shimei Wan, Shasha Li, Xiaoci Wang, Xiqiao Zhou, Yue Zhao

**Affiliations:** ^1^ Department of Endocrinology, Affiliated Hospital of Nanjing University of Chinese Medicine, Jiangsu Province Hospital of Chinese Medicine, Nanjing, China; ^2^ Department of Ophthalmology, Affiliated Hospital of Nanjing University of Chinese Medicine, Jiangsu Province Hospital of Chinese Medicine, Nanjing, China; ^3^ Department of General Internal Medicine, Affiliated Hospital of Nanjing University of Chinese Medicine, Jiangsu Province Hospital of Chinese Medicine, Nanjing, China; ^4^ The First College of Clinical Medicine, Nanjing University of Chinese Medicine, Nanjing, China

**Keywords:** hyperoside, diabetic retinopathy, retinal endothelial cells, TGF-β1, miR-200b, VEGF

## Abstract

**Aims:**

This study aimed to evaluate the role of hyperoside in ameliorating the retinal injury of diabetic retinopathy (DR) rats and the dysfunction of retinal endothelial cells (RECs) in high glucose.

**Methods:**

RECs were cultured in various groups including high glucose and different concentrations of hyperoside. The viability, migration, and tube formation of RECs and the expression of transforming growth factor-beta 1 (TGF-β1)/micro-RNA 200b (miR-200b)/vascular endothelial growth factor (VEGF) in each group were assayed. Sprague–Dawley rats were used for DR modeling and were treated with hyperoside. The tissue pathology of the rat retina and the expression of TGF-β1/miR-200b/VEGF in the retinal tissues of each group were examined.

**Results:**

Excessive proliferation, migration, and tube formation of RECs were induced by high glucose. The retinal pathological changes and vasculopathy in DR rats were more serious compared with those in normal rats. The expression levels of TGF-β1 and VEGF in the high glucose-induced REC group and in DR rat retina were markedly upregulated, but those of miR-200b were noticeably downregulated. However, hyperoside could significantly inhibit the high glucose-induced overproliferation, migration, and tube formation of RECs, alleviate the retinal injury in DR rats, and reverse the expression of TGF-β1/miR-200b/VEGF in the high glucose-induced REC and DR rat retina, dose-dependently.

**Conclusions:**

Hyperoside could ameliorate retinal injury in DR rats and the high glucose-induced dysfunction of RECs by regulating the TGF-β1/miR-200b/VEGF pathway.

## Highlights

Our previous study has provided proof that *Abelmoschus manihot* can inhibit the progression of diabetic retinopathy (DR), but the specific mechanism is still unclear. Hyperoside is a primary active ingredient of *A. manihot* and is one of the flavonoid glycosides with anti-inflammatory and antioxidant effects. Therefore, hyperoside was used to interfere with DR rats and the high glucose-cultured RECs in this study. The results suggest that hyperoside could ameliorate the retinal injury in DR rats and the high glucose-induced dysfunction of retinal endothelial cells (RECs) by regulating the TGF-β1/miR-200b/VEGF pathway. The potential mechanism of *A. manihot* against DR was preliminarily proven. This is beneficial for the further exploration of herbal medicine in the treatment of DR.

## 1 Introduction

Diabetic retinopathy (DR) is one of the major microvascular complications of diabetes mellitus (DM) and is the leading cause of vision loss in middle-aged and elderly people around the world (1). A global meta-analysis including 59 studies has shown prevalence rates of 22.27% for DR, 6.17% for vision-threatening DR, and 4.07% for clinically significant macular edema among individuals with DM (2). With its growing prevalence and prolonged duration, DR has brought great harm and burden to human health and the medical care system (3). Therefore, there is a need to explore effective treatments for and the relative mechanism of DR in order to prevent its progression. According to the Diabetic Retinopathy Preferred Practice Pattern Guideline (version 2019) issued by the American Academy of Ophthalmology (4), healthy lifestyle and strict control of blood glucose, blood pressure, and serum lipids are beneficial to the prevention of DR. However, the progression of diabetic complications appears more than hard to control. The validity of several anti-DR drugs such as alprostadil, antioxidants, anti-thrombotics, and protein kinase C inhibitors needs further evaluation from large-sample, multicenter clinical research. To solve this dilemma, a number of complementary and alternative treatments via traditional herbal medicine have been tentatively used for DR. Interestingly, more and more herbal medicines have shown potential and promising efficacy against DR (5).

The recent clinical study by our team indicated that *Abelmoschus manihot* could improve the severity of DR, the Early Treatment Diabetic Retinopathy Study (ETDRS) vision scores, and the macular edema in type 2 diabetes. In addition, the specific therapeutic mechanism appears to be associated with inhibition of the vascular endothelial growth factor (VEGF) levels, which needs further investigation (6). Previous studies have shown that the activation of the transforming growth factor-beta 1 (TGF-β1)/micro-RNA 200b (miR-200b)/VEGF pathway by hyperglycemia might play an essential role in the destruction of the blood–retinal barrier (BRB) and in the pathogenesis of DR (7). Related studies have also shown that hyperoside is one of the main active ingredients of *A. manihot*, which could inhibit the expression of TGF-β1 in a rat model of diabetic nephropathy (8). Whether hyperoside also plays a role in the treatment of DR by improving the TGF-β1/miR-200b/VEGF pathway needs further study. Therefore, the present study was primarily focused on the effects of hyperoside on the pathological process of DR, including the retinal injury in diabetic rats, the proliferation of retinal endothelial cells (RECs), and the regulation of the TGF-β1/miR-200b/VEGF pathway in high glucose (HG). Moreover, we hoped to explore the preliminary effect and mechanism of hyperoside in the treatment of DR.

## 2 Materials and methods

### 2.1 Hyperoside

Hyperoside extracted from *A. manihot* was purchased from the agent company (MB6720; Meilunbio, Guangzhou, China). Hyperoside (Chemical Abstracts Service no. 482-36-0; 2) appears as a yellow crystalline powder, which was stored in a cool, dry, air-proof and innocuous condition with limited light and heat, and its purity, as assayed by high-performance liquid chromatography, was 99.45%.

### 2.2 Cell culture and treatments

The vial for the primary human RECs was purchased from CellSystems (ACBRI-181; Troisdorf, Germany). RECs were cultured in complete growth medium consisting of 89% low-glucose Dulbecco’s modified Eagle’s medium (DMEM; 11885092; Gibco, Waltham, MA, USA), 10% fetal bovine serum (FBS; 10099141, Gibco), and 1% antibiotics (100 U/ml penicillin and 100 µg/ml streptomycin) at 37°C in a humidified atmosphere of 5% CO_2_. When the RECs were cultured to the logarithmic growth phase (approximately 90% confluence), the medium was aspirated. The cells were digested with 0.25% trypsin, and the cell suspension was collected and centrifuged at 1,000 rpm for 3 min, after which the supernatant was discarded. Fresh medium was added to resuspend the cells. A small aliquot of the cell suspension was taken for microscopic counting, and the cell density was adjusted to the desired concentration based on the count. Subsequently, the cells were seeded or transferred into new culture plates. The cell culture experiments consisted of three parts, as follows:

1. The sub-cultured RECs were randomly divided into five groups with different glucose concentrations in the culture medium: the normal glucose (NG) group (5 mmol/L glucose); the high-glucose-1 (HG-1) group (20 mmol/L glucose); the high-glucose-2 (HG-2) group (25 mmol/L glucose); the high-glucose-3 (HG-3) group (30 mmol/L glucose); and the high-glucose-4 (HG-4) group (35 mmol/L glucose). Differences in the proliferative activity of RECs among these five groups were compared, and the consistency of these differences was repeatedly observed across distinct incubation periods (24, 48, and 72 h).
2. The sub-cultured RECs were also randomly assigned into five other groups: the NG group (5 mmol/L glucose); the high-glucose (HG) group (35 mmol/L glucose); the mannitol (MT) group (5 mmol/L glucose plus 30 mmol/L mannitol as an osmotic pressure control); the HG + 100 µg/ml hyperoside (HG+H100) group; and the HG + 400 µg/ml hyperoside (HG+H400) group. Hyperoside was dissolved in 0.1% dimethyl sulfoxide (DMSO) solution for REC treatment. The effects of a HG environment on the proliferative activity of RECs were elucidated, along with the impact of hyperoside on these effects.
3. The sub-cultured RECs were randomly divided into six other groups: the NG group (5 mmol/L glucose); the NG + miR-200b inhibitor (NG+MI) group (NG plus an miR-200b inhibitor and a transfection reagent); the NG + normal control (NG+NC) group (NG plus a transfection reagent only); the HG group (35 mmol/L glucose); the HG + miR-200b mimic (HG+MM) group (HG plus an miR-200b mimic and a transfection reagent); and the HG + normal control (HG+NC) group (HG plus a transfection reagent only). The role of the TGF-β1/miR-200b/VEGF pathway in HG-induced excessive proliferation of RECs was preliminarily explored through mimic/inhibitor experiments.

### 2.3 Cell Counting Kit-8 assay

The RECs were adjusted to a cell suspension with a density of 6 × 10^4^/ml. A 96-well cell culture plate was prepared, and each well was labeled according to the experimental groups described in *Section 2.2*, with duplicate wells set up for each group. Each well was loaded with 100 μl of the cell suspension (equivalent to 6 × 10^3^ cells per well). In addition, blank control wells were included, which contained equal volumes of cell-free basal medium. The plate was incubated in a 37°C, 5% CO_2_ incubator for 24 h. After incubation, 100 μl of medium containing different concentrations of glucose or mannitol and 0.2 μl hyperoside solution (prepared in 0.1% DMSO) was added to each corresponding well. This resulted in final hyperoside concentrations of 100 and 400 μg/ml in the respective wells. The plate was then returned to the 37°C, 5% CO_2_ incubator for an additional 24, 48, or 72 h. Finally, the cells were observed and photographed under a ×40 microscope. Subsequently, the RECs were incubated with 10 µl Cell Counting Kit-8 (CCK-8) agent (CK04; Dojindo, Kumamoto, Japan) for 3 h. The optical density at 450 nm of each well was determined using a microplate reader (PerkinElmer EnSpire, Waltham, MA, USA) to calculate the relative viability of the RECs.

### 2.4 Transwell assay

The RECs (2 × 10^4^ cells per well) and various concentrations of glucose and hyperoside media were respectively added to the upper chamber of 24-well Transwell inserts (8-μm pore size; 14341, Labselect, Hefei, China). For the lower chamber, the medium containing 20% FBS and 5 mmol/L glucose was added, and the follow-up culture was performed at 37°C for 24 h. The RECs at the bottom of the upper chamber were stained with 0.5% crystal violet, and the cells inside the upper chamber were removed with a cotton swab. The RECs outside the bottom of the upper chamber were observed and counted under a light microscope (CKX31; Olympus, Tokyo, Japan) at ×200 magnification in five random visual fields.

### 2.5 Cell tube formation assay

A Matrigel matrix glue (10 mg/ml; 356234, Corning, Corning, NY, USA) was slowly injected into a 96-well plate placed in an ice bath with a pre-cooled pipette. The volume of injection in each well was 100 μl. The Matrigel matrix glue was then placed in an incubator at 37°C for 30 min to solidify the glue. Afterward, the cultured cells were inoculated into the 96-well plate with 1 × 10^4^ cells per well. The different media described above were respectively added into the plate and then continuously cultured for another 6 h. The tubular structure was recorded using a light microscope (CKX31, Olympus), and the branch points were visualized and calculated in five random regions.

### 2.6 Cell transfection

The miR-200b mimic and inhibitor were purchased from GenePharma (Shanghai, China) and transfected into the RECs according to the manufacturer’s instructions. Firstly, the cells were seeded in complete medium for 24 h to grow to 30% confluence before transfection. Thereafter, DMEM without serum was used to dilute the miR-200b mimic/inhibitor and the transfection reagent (R4000; Engreen, Beijing, China). Subsequently, these two diluents were mixed and kept stable for 15 min. The mixed liquid was added to the RECs with complete medium. After 24 h of incubation in a 5% CO_2_ humidified atmosphere at 37°C, the transfection medium could be replaced with NG or HG complete medium for another 24 h before subsequent experiments.

### 2.7 Animals

A total of 40 specific pathogen-free healthy male Sprague–Dawley (SD) rats (5–6 weeks old), weighing from 170 to 200 g, were purchased from Qing Long Shan Animal Centre (Nanjing, China). These rats were treated and operated on according to the guidelines of the Animal Ethics Committee of the Affiliated Hospital of Nanjing University of Chinese Medicine (ethical approval no. 2020DW-21-02). Rats were housed at constant room temperature (20–22°C) and relative humidity (50%–60%) under a controlled 12-h light/dark cycle and had free access to water and the standard laboratory diet.

### 2.8 DR rat model and drug treatment

We randomly selected eight SD rats as the NC group, with the other 32 rats used to establish DR models, which were randomly divided into four groups including the DR group (DR model with no treatment), the DR + low-dose hyperoside (DR+L-HY) group (DR model with 6.5 mg hyperoside per 1 kg rat body mass per day, 6.5 mg kg^−1^ day^−1^), the DR + high-dose hyperoside (DR+H-HY) group (DR model with 19.5 mg kg^−1^ day^−1^ hyperoside), and the DR + calcium dobesilate (DR+CD) group (DR model with 135 mg kg^−1^ day^−1^ calcium dobesilate). DR models were established as follows:

1. After 4 weeks of high-fat diet (rodent diet with 45% calories from fat), SD rats were fasted for 12 h and then intraperitoneally injected with 1% streptozotocin (STZ; V900890, Sigma-Aldrich, St. Louis, MO, USA) solution at a dose of 25 mg/kg and injected again with the same dose after 2 days. The random blood glucose of each SD rat was measured at 72 h after injection. When all blood glucose levels were >16.7 mmol/L, then DM models were considered successful.
2. DM rats were allowed to eat and drink normally. Their blood glucose and body mass (BM) were measured regularly, and morphological changes were observed throughout the study. After the 8-week continuous feeding, one NC and two DM rats were randomly selected and their retinal tissue pathology and trypsin digest examined to evaluate successful DR models. After establishment of the DR models, the DR+L-HY, DR+H-HY, and DR+CD were given respective doses of hyperoside and calcium dobesilate, which were dissolved in 0.5% sodium carboxymethyl cellulose solution by gavage. On the other hand, the NC and DR groups were given an equal amount of 0.5% sodium carboxymethyl cellulose solution. All rats were continuously given food, water, and drugs for 8 weeks and then anesthetized to death. The eyeballs were isolated and made into optic cups for further experiments.

### 2.9 Retinal pathology

Following enucleation of the rat eyeballs, intact globes containing optic cup–retinal complexes were first fixed in 4% paraformaldehyde for 24 h. Subsequently, the cornea, lens, and vitreous body were removed, retaining only the fixed optic cup–retinal complex. The adhesion points between the retina and the inner cup wall (primarily located at optic disc margins) were gently dissected under a stereomicroscope [in 4°C phosphate-buffered saline (PBS)] using Dumont #5 microforceps. The retinal tissues were then dehydrated in a concentration gradient of alcohol, made transparent with xylene, and then paraffin-embedded. The paraffin-embedded tissue blocks were cut into 4-μm-thick sections. After xylene dewaxing and gradient alcohol rehydration, the tissue sections were stained with a hematoxylin–eosin (H&E) staining solution (BL-700A; BioSharp, Tallinn, Estonia). Pathological changes of the retinal tissues in all groups were observed under a light microscope (CKX31, Olympus) after the tissue sections were dehydrated by gradient alcohol, made transparent via xylene, and sealed with neutral resin.

### 2.10 Retinal trypsin digest

To analyze the vasculopathy for retinal capillary degeneration, REC proliferation, and retinal pericyte (RPC) loss, the retinal trypsin digest technique was used as follows: The retinal tissues were isolated and digested in 3% trypsin solution (25200-072, Gibco) at 37°C for 2 h, and the dissolved retina was transferred into distilled water with a glass rod and gently shaken to wash away the inner boundary membrane and the residual retinal nerve tissue. Afterward, the retinal vascular network, which had been fully digested and separated, was lifted with the glass rod and quickly transferred into a glass slide for full spreading and natural drying. The slides were stained with periodic acid–Schiff (PAS; G1285, SolarBio, Beijing, China) and observed under a microscope (CKX31, Olympus). The retinal vascular quantity (RVQ) and the endothelial cell-to-pericyte ratio (E/P) in all groups were counted and compared under the microscope. RVQ was calculated as follows: At ×200 magnification, five regions of each rat’s retinal vascular network were randomly selected, and the number of capillaries passing through the transverse and the vertical diameter of the center of each region were calculated and averaged, with the mean number of five regions taken as the RVQ of this sample. The calculation of E/P was similar to that for RVQ: At ×400 magnification, five visual fields of each rat’s retinal vascular network were randomly selected, and the number and ratio of RECs and pericytes in each visual field were calculated, with the mean value of five visual fields used as the E/P value of the rat.

### 2.11 Retinal immunofluorescence detection

The retinal tissue sections were deparaffinized and rehydrated, followed by antigen repair for 15 min and goat serum sealing for 20 min. Subsequently, the sections were incubated with TGF-β1 primary antibody (SAB4502954, Sigma) at 4°C for 12 h, followed by incubation with secondary antibody with FITC (16868; AAT Bioquest, Pleasanton, CA, USA) at 37°C in darkness for 30 min. After washing with PBS, each section was added with DAPI (D9542, Sigma-Aldrich) and incubated at 37°C in darkness for 5 min. Finally, the expression of the TGF-β1 protein in the retinal tissue section was observed under a fluorescence microscope (BX43, Olympus). The expression of the VEGF protein in retinal tissue was determined with specific antibodies using a similar process.

### 2.12 Western blotting assay

After measuring the concentrations of total proteins from retinal tissues or the REC lysates with the BCA protein assay kit (ab102536; Abcam, Cambridge, UK), the proteins were separated by SDS-PAGE and then transferred into PVDF membranes (1620177; Bio-Rad, Hercules, CA, USA). Subsequently, the membranes were blocked with 5% skimmed milk for 1 h, followed by incubation with TGF-β1 rabbit antibody (3709S; Cell Signaling Technology, Danvers, MA, USA) for 14 h at 4°C and goat anti-rabbit IgG–HRP (A0545, Sigma) for 1 h at 37°C. The immune complex was detected with enhanced chemiluminescence using the ECL kit (wbklso500; Merck Millipore, Burlington, MA, USA). After exposure and development, the gray value of each band was quantified, and the relative levels of TGF-β1 protein to β-actin were determined with an imaging system (ImageQuant LAS 4000). The VEGF protein expression was detected with the corresponding primary and secondary antibodies using the above-mentioned methods.

### 2.13 Quantitative real-time reverse transcription polymerase chain reaction analysis

Total RNA was extracted from retinal tissues or RECs using an RNA extraction kit (15596018; ThermoFisher, Waltham, MA, USA) and subjected to reverse transcription with a reverse transcription kit (4368814; Invitrogen, Waltham, MA, USA). The samples were amplified by DNA polymerase (11146173001; Roche, Basel, Switzerland) using specific primers by quantitative reverse transcription PCR (qRT-PCR) system (Applied Biosystems 7500). The relative expression levels of miRNAs (miR-200b) and mRNAs (*TGFB1/Tgfb1* and *VEGFA/Vegfa*) normalized by U6 small nuclear RNA and β-actin mRNA, respectively, were calculated with the 2^−ΔΔCt^ method.

### 2.14 Statistical analysis

All experiments were repeated three times. The results are expressed as the mean ± standard deviation. Differences among groups were statistically analyzed using one-way/two-way ANOVA with the GraphPad Prism 8 software. A *p* < 0.05 was considered significantly different.

## 3 Results

### 3.1 High glucose induced excessive proliferation of RECs

**Figure 1:**
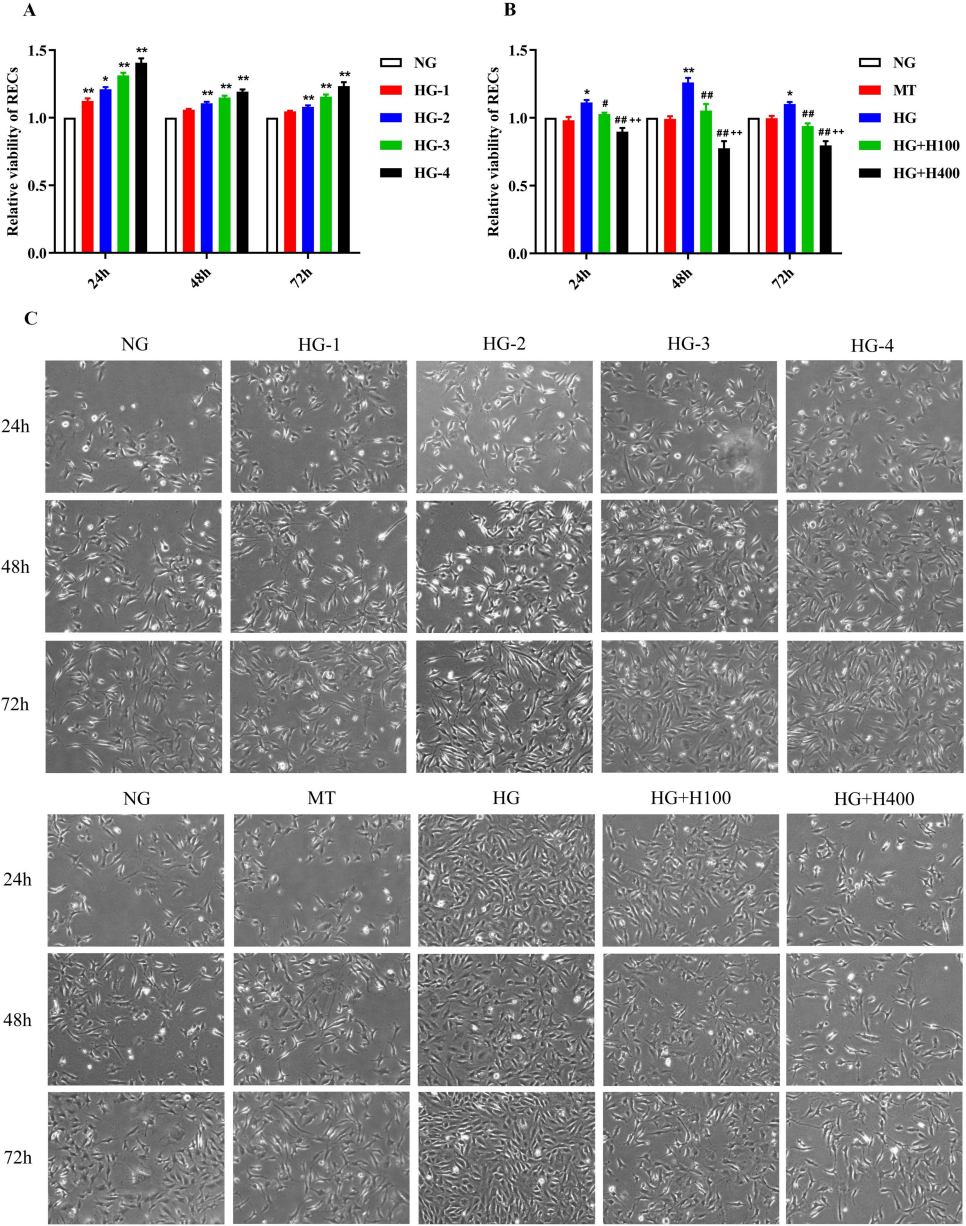
The viability of retinal endothelial cells (RECs) was positively correlated with glucose concentration and was noticeably inhibited by hyperoside. **(A)** Relative viability of RECs in different concentrations of glucose measured using CCK-8 after 24, 48, and 72 h of incubation. **(B)** Relative viability of RECs in normal glucose (NG), mannitol (MT), high glucose (HG), HG with 100 µg/ml hyperoside (HG+H100), and HG with 400 µg/ml hyperoside (HG+H400) measured using CCK-8 after 24, 48, and 72 h of incubation. RECs in HG+H100 and HG+H400 were transferred into the HG medium and added with 100 and 400 µg/ml hyperoside, respectively, for further incubation. **(C)** Representative images of the RECs in different groups under a microscope at ×40 magnification. All CCK-8 tests were repeated more than three times for statistical analysis. Data are presented as the mean ± standard deviation. A *p* < 0.05 is statistically significant. *p < 0.05 *vs*. NG; **p < 0.01 *vs*. NG; ^#^ *p* < 0.05 *vs*. HG; ^##^ *p* < 0.01 *vs*. HG; ^++^ *p* < 0.01 *vs*. HG+H100.

To evaluate the effect of HG on the proliferation of RECs, the RECs were cultured with different concentrations of glucose and their viability measured at varied incubation times, which were compared with those of the NG group. As shown in **Figures 1A, C**, the viability of RECs in HG-1 was significantly elevated compared with that in NG at the 24-h period (1.12 ± 0.04 *vs*. 1.00 ± 0.00, *p* < 0.01). Similarly, the viability of RECs in HG-2 was also greatly elevated compared with that in NG at the 24-, 48-, and 72-h periods (1.21 ± 0.04 *vs*. 1.00 ± 0.00, *p* < 0.01; 1.11 ± 0.02 *vs*. 1.00 ± 0.00, *p* < 0.01; and 1.08 ± 0.03 *vs*. 1.00 ± 0.00, *p* < 0.01, respectively). For other comparisons, such as for HG-3 *vs*. NG and HG-4 *vs*. NG at the 24-, 48-, and 72-h periods, all of the findings suggest that the viability of RECs was significantly related to the increase of glucose concentration. These results indicate that HG could induce the excessive proliferation of RECs.

### 3.2 Hyperoside inhibited the excessive proliferation of RECs in high glucose

The effects of hyperoside were evaluated against the HG-induced excessive viability of RECs. As presented in **Figures 1B, C**, all of the REC viability levels in HG at different incubation periods (24, 48, and 72 h) were higher than those of the NG groups (1.11 ± 0.04 *vs*. 1.00 ± 0.00, *p* < 0.05; 1.26 ± 0.07 *vs*. 1.00 ± 0.00, *p* < 0.01; and 1.10 ± 0.03 *vs*. 1.00 ± 0.00, *p* < 0.05, respectively). On the other hand, all of the REC viability levels in HG+H100 at the different treatment periods were lower than those of the HG groups (1.03 ± 0.02 *vs*. 1.11 ± 0.04, *p* < 0.05; 1.05 ± 0.11 *vs*. 1.26 ± 0.07, *p* < 0.01; and 0.94 ± 0.04 *vs*. 1.10 ± 0.03, *p* < 0.01, respectively). A similar difference was also observed in the comparisons of HG+H400 *vs*. HG and HG+H400 *vs*. HG+H100. The results showed that both the low-concentration (100 µg/ml) and high-concentration (400 µg/ml) hyperoside could significantly inhibit the viability of RECs under a HG condition, and the stronger inhibition of REC viability occurred along with the increase of hyperoside concentration. These data suggest that hyperoside could dose-dependently inhibit the excessive proliferation of RECs in HG.

### 3.3 Hyperoside inhibited the excessive migration and tube formation of RECs in high glucose

**Figure 2:**
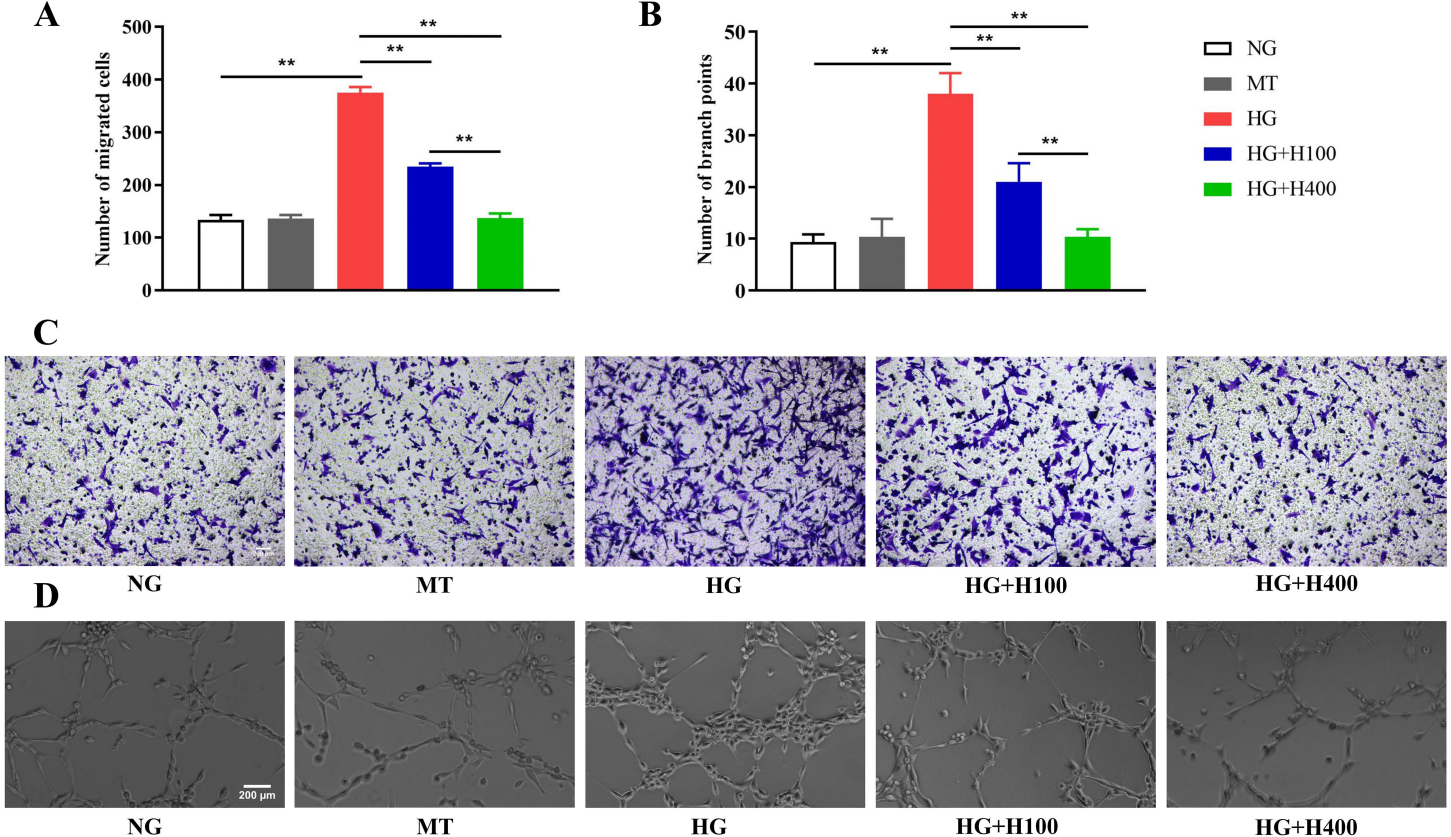
The excessive migration and the tube formation of retinal endothelial cells (RECs) in high glucose **(HG)** were inhibited by hyperoside. **(A)** Comparison of the migrated RECs measured by the Transwell assay in HG and different concentrations of hyperoside. **(B)** Comparison of the REC branch points assessed using the tube formation assay in HG and different concentrations of hyperoside. **(C)** Representative photographs of the Transwell assay in different groups under a microscope (×200). **(D)** Representative photographs of the tube formation assay in different groups under a microscope (×200). All Transwell and tube formation tests were repeated three times for statistical analysis. Data are presented as the mean ± standard deviation. A *p* < 0.05 is statistically significant. **p < 0.01.

It is well known that DR may display not only REC proliferation but also a significantly increased REC migration and tube formation. Therefore, the role of hyperoside in the migration and tube formation of RECs was assessed in HG. As shown in **Figures 2A, C**, the number of migrated RECs in HG was significantly higher than that in NG (375.7 ± 10.4 *vs*. 133.3 ± 9.6, *p* < 0.01) and also higher than those in HG+H100 and HG+H400 [375.7 ± 10.4 *vs*. 234.7 ± 6.5 (*p* < 0.01) and 375.7 ± 10.4 *vs*. 137.0 ± 9.0 (*p* < 0.01), respectively]. Similar results were also observed in the tube formation assay, as presented in **Figures 2B, D**, with the number of branch points of RECs in HG being significantly higher than that in NG (38.0 ± 4.0 *vs*. 9.3 ± 1.5, *p* < 0.01). In contrast, the numbers in HG+H100 and HG+H400 were significantly decreased compared with that in HG [21.0 ± 3.6 *vs*. 38.0 ± 4.0 (*p* < 0.01) and 10.3 ± 1.5 *vs*. 38.0 ± 4.0 (*p* < 0.01), respectively]. These data suggest that hyperoside could inhibit the excessive migration and tube formation of RECs induced by HG.

### 3.4 TGF-β1/miR-200b/VEGF pathway contributed to the overproliferation of RECs in high glucose

**Figure 3:**
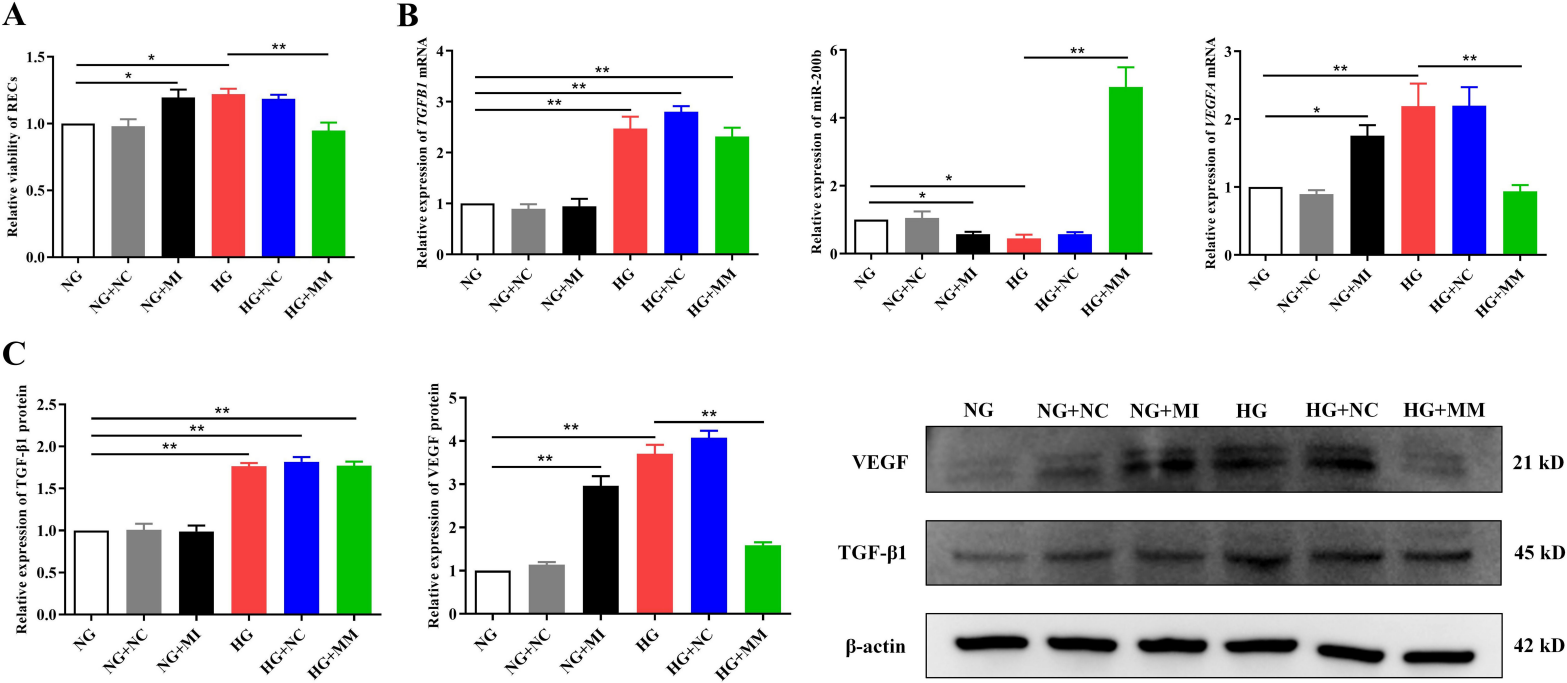
The TGF-β1/miR-200b/VEGF pathway played a key role in the high glucose-induced proliferation of retinal endothelial cells (RECs). **(A)** Viability of the RECs in all groups measured by CCK-8 after transfection. **(B)** Expression of the *TGFB1* and *VEGFA* mRNA and miR-200b in the different groups assayed using quantitative reverse transcription PCR (qRT-PCR) after transfection. **(C)** Expression of the TGF-β1 and VEGF proteins in the different groups detected using Western blotting after transfection. The CCK-8, PCR, and Western blotting tests were repeated three or more times for statistical analysis. Data are presented as the mean ± standard deviation. A *p* < 0.05 is statistically significant. *p < 0.05, **p < 0.01.

To determine the role of the TGF-β1/miR-200b/VEGF pathway in the overproliferation of RECs induced by HG, we transfected the miR-200b mimic and the miR-200b inhibitor into RECs in the HG and NG groups, respectively. As shown in **Figure 3**, the miR-200b inhibitor in NG+MI could significantly elevate the viability of RECs (1.20 ± 0.15 *vs*. 1.00 ± 0.00, *p* < 0.05) (**Figure 3A**) and the expression of the *VEGFA* mRNA and protein [1.76 ± 0.27 *vs*. 1.00 ± 0.00 (*p* < 0.05) and 2.96 ± 0.39 *vs*. 1.00 ± 0.00 (*p* < 0.01) respectively] (**Figures 3B, C**), but reduced the miR-200b expression (0.57 ± 0.12 *vs*. 1.00 ± 0.00, *p* < 0.05) (**Figure 3B**) compared with NG. However, the miR-200b mimic in HG+MM could significantly downregulate the viability of RECs (0.95 ± 0.15 *vs*. 1.22 ± 0.10, *p* < 0.01) (**Figure 3A**) and the *VEGFA* mRNA and protein levels [0.94 ± 0.16 *vs*. 2.19 ± 0.58 (*p* < 0.01) and 1.59 ± 0.13 *vs*. 3.70 ± 0.35 (*p* < 0.01) respectively] (**Figures 3B, C**), but upregulated the miR-200b expression (4.91 ± 1.00 *vs*. 0.45 ± 0.18, *p* < 0.01) (**Figure 3B**) compared with HG. There were no significant differences in the *TGFB1* mRNA and protein expressions between NG and NG+MI. Moreover, there were no significant differences in the *TGFB1* mRNA and protein expressions between HG and HG+MM. The expressions of the *TGFB1* mRNA and protein were notably enhanced by HG, but were not regulated by the miR-200b mimic or the inhibitor (**Figures 3B, C**). These results indicate that the HG-induced activation of the TGF-β1/miR-200b/VEGF pathway played a positive role in the excessive proliferation of RECs.

### 3.5 Hyperoside regulated the TGF-β1/miR-200b/VEGF pathway in high glucose-cultured RECs

**Figure 4:**
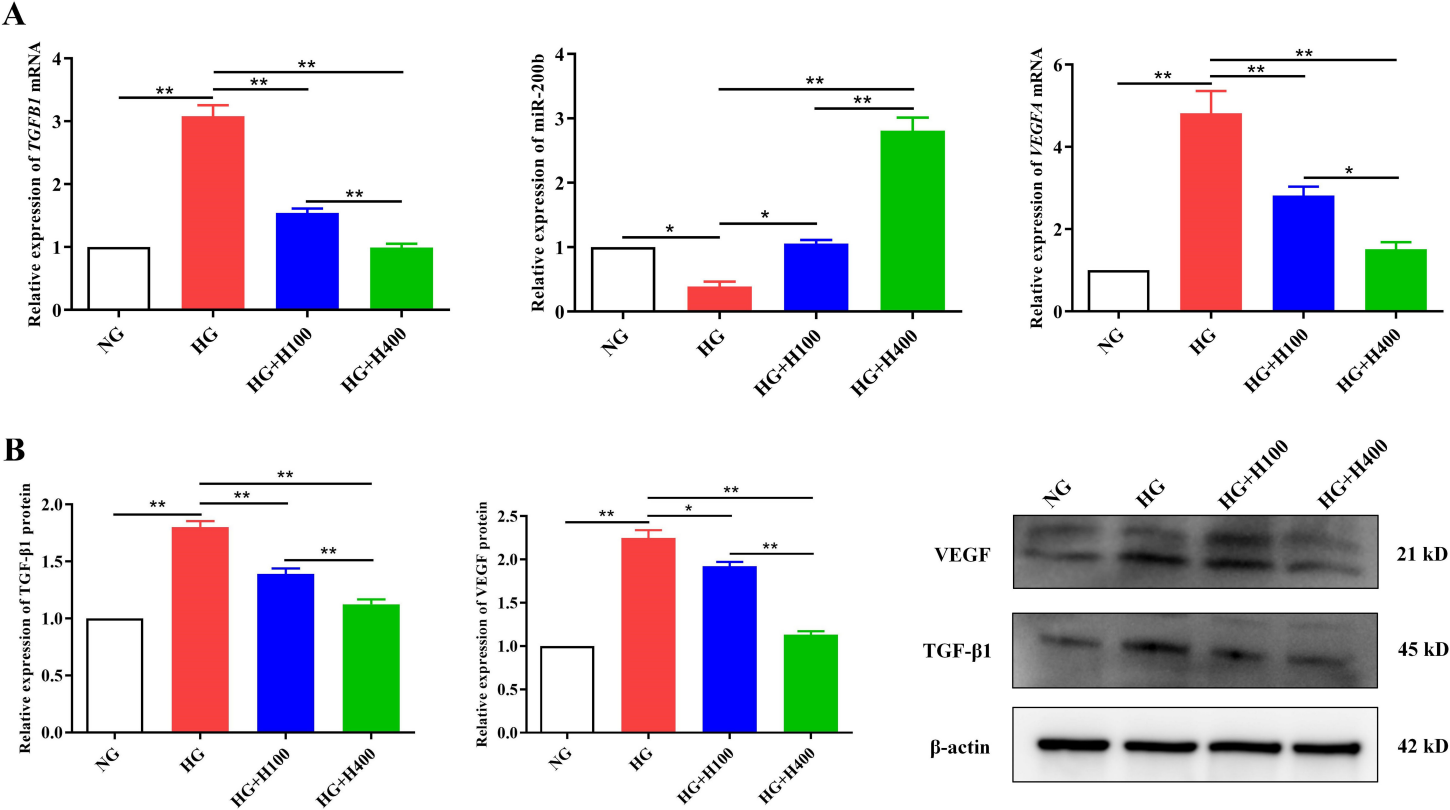
Hyperoside downregulated the TGF-β1 and VEGF and upregulated the miR-200b of retinal endothelial cells (RECs) in high glucose (HG). **(A)** Expression of the *TGFB1* and *VEGFA* mRNA and miR-200b in different groups examined with quantitative reverse transcription PCR (qRT-PCR). **(B)** Expression of the TGF-β1 and VEGF proteins in different groups detected using Western blotting. Both the PCR and Western blotting tests were repeated three or more times for statistical analysis. Data are presented as the mean ± standard deviation. A *p*<0.05 is statistically significant. *p < 0.05, **p < 0.01.

To elucidate the protective mechanism of hyperoside against the overproliferation of RECs in HG, variations in the TGF-β1, miR-200b, and VEGF levels in the different groups were analyzed. Compared with NG, HG could remarkably induce high mRNA and protein expressions of *TGFB1* and *VEGFA* [3.08 ± 0.35 *vs*. 1.00 ± 0.00 (*p* < 0.01) and 1.80 ± 0.09 *vs*. 1.00 ± 0.00 (*p* < 0.01); 4.82 ± 1.08 *vs*. 1.00 ± 0.00 (*p* < 0.01) and 2.25 ± 0.16 *vs*. 1.00 ± 0.00 (*p* < 0.01)] (**Figures 4A, B**) and noticeably inhibit the miR-200b expression (0.39 ± 0.13 *vs*. 1.00 ± 0.00, *p* < 0.05) (**Figure 4A**). However, hyperoside reversed the expressions of TGF-β1/miR-200b/VEGF under the HG condition. As presented in **Figure 4**, the low concentration of hyperoside in HG+H100 could significantly inhibit the mRNA and protein levels of *TGFB1* and *VEGFA* [1.54 ± 0.14 *vs*. 3.08 ± 0.35 (*p* < 0.01) and 1.39 ± 0.08 *vs*. 1.80 ± 0.09 (*p* < 0.01); 2.82 ± 0.43 *vs*. 4.82 ± 1.08 (*p* < 0.01) and 1.92 ± 0.09 *vs*. 2.25 ± 0.16 (*p* < 0.05) respectively] (**Figures 4A, B**) and upregulate the miR-200b level (1.06 ± 0.10 *vs*. 0.39 ± 0.13, *p* < 0.05) (**Figure 4A**) compared with HG. A similar difference was also observed in the comparisons of HG+H400 *vs*. HG and HG+H400 *vs*. HG+H100. There was a stronger regulation of the TGF-β1/miR-200b/VEGF pathway when the hyperoside concentration was increased. Therefore, these data indicate that hyperoside could decrease TGF-β1 and VEGF, but increase miR-200b in RECs under a HG condition in a dose-dependent manner.

### 3.6 BM and fasting blood glucose of rats in the different groups

**Figure 5:**
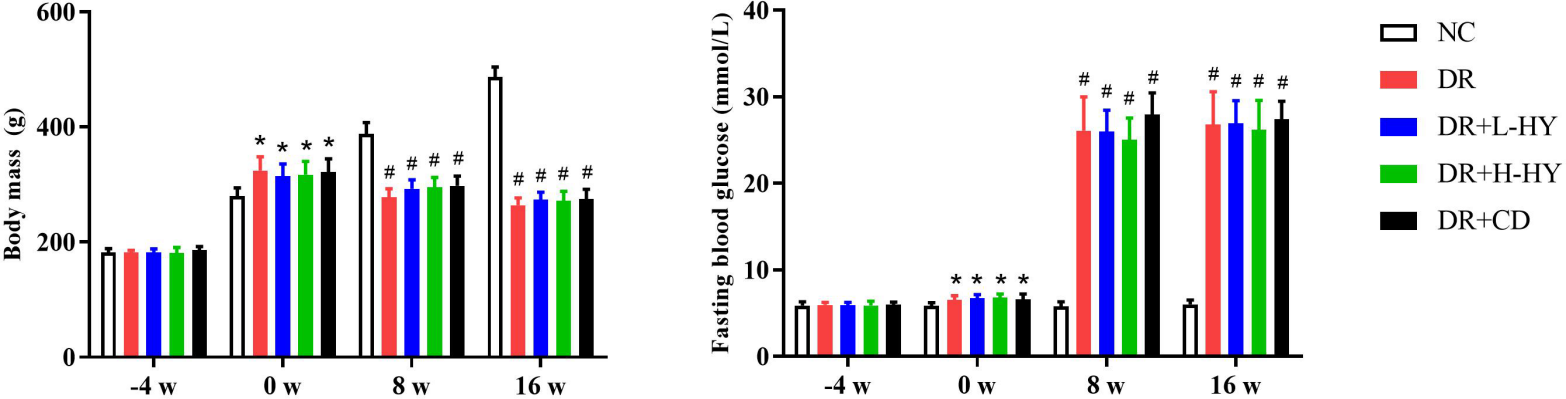
Comparison of the body mass (BM) and fasting blood glucose (FBG) of rats at different times. The BM and FBG of rats were both measured on fasting in the morning. There were six rats in each group. Data are presented as the mean ± standard deviation. A *p* < 0.05 is statistically significant. *p<0.05 *vs*. normal control (NC) on the same week; ^#^ *p* < 0.01 *vs*. NC on the same week.

We assessed the BM and fasting blood glucose (FBG) of rats at different periods: 4 weeks before STZ injection (−4 w), 0 week before STZ injection (0 w), 8 weeks after STZ injection (8 w), and 16 weeks after STZ injection (16 w). As shown in **Figure 5**, there were no significant differences in the BM and FBG at −4 w in all groups. After 4 weeks of high-fat diet, the BM and FBG in all DR groups (DR, DR+L-HY, DR+H-HY, and DR+CD) were significantly higher than those in NC. At 8 w and 16 w, the BM values in all DR groups were significantly lower than those in NC, while the FBG levels were significantly higher than those in NC. However, there were no significant differences in the BM and FBG in all DR groups at any time. It could be speculated that the primary effect of hyperoside and calcium dobesilate is not aimed at hyperglycemia.

### 3.7 Hyperoside improved the pathology of retinal tissue

**Figure 6:**
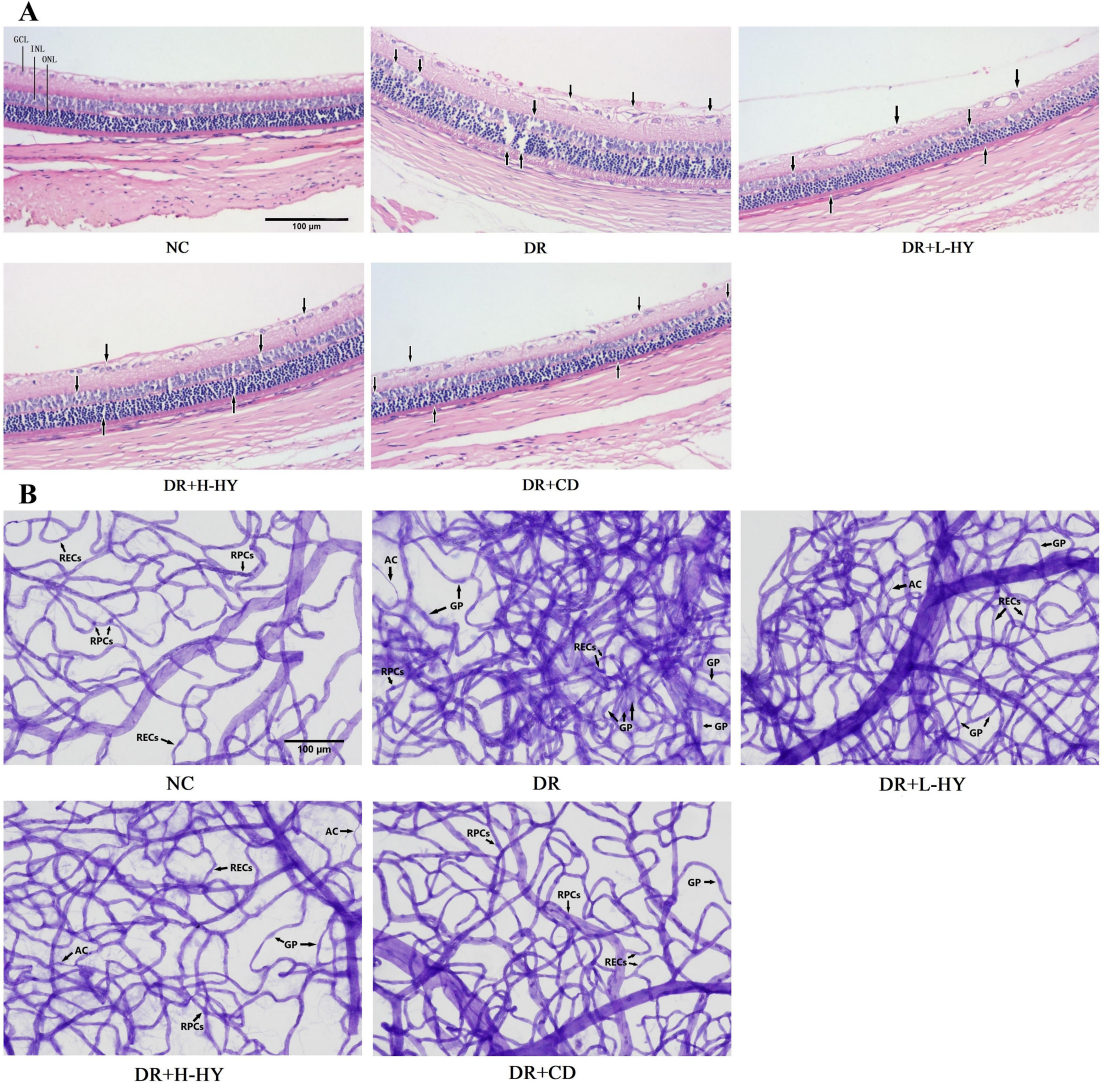
Representative pathological changes of the retinal tissues and typical images of retinal vasculopathy in the different groups. **(A)** Pathological changes in the retinal tissue detected using hematoxylin and eosin (H&E) staining (×100). **(B)** Retinal vasculopathy detected with retinal trypsin digest and periodic acid–Schiff (PAS) staining (×200). *GCL*, ganglion cell layer; *INL*, inner nuclear layer; *ONL*, outer nuclear layer; *RECs*, retinal endothelial cells; *RPCs*, retinal pericytes; *AC*, acellular capillaries; *GP*, ghost pericytes. Differences in the pathological changes and the retinal vasculopathy in all groups are indicated by *black arrows*.

To further analyze the possible role of hyperoside on retinal injury in diabetic rats, the pathological changes of the retinal tissues were assessed in the NC, DR, DR+L-HY, DR+H-HY, and DR+CD groups. As shown in **Figure 6A**, in the NC group, the ganglion cell layer (GCL) in retinal tissue was orderly, and the inner and outer nuclear layers (INL and ONL, respectively) were closely arranged. In the DR group, the arrangement of the GCLs was disordered, and the INL and ONL appeared sparser and less compact than those in NC. However, these pathological changes were alleviated in the DR+L-HY group and improved in the DR+H-HY and DR+CD groups, marked by black arrows in **Figure 6A**. These results indicate that hyperoside could alleviate retinal tissue damage in DR rats.

### 3.8 Hyperoside improved retinal vasculopathy

To confirm the improvement of hyperoside on diabetic retinal injury, its effect on the retinal vessels in DR rats was also evaluated. Retinal trypsin digest and retinal vascular staining were performed in the NC, DR, DR+L-HY, DR+H-HY, and DR+CD groups. In the NC group, the retinal capillaries were regularly distributed with smooth vascular branches, uniform diameter, and very few acellular capillaries (AC). In the DR group, the retinal capillary network remained dense, disorderly, and twisted, with uneven diameter, more AC, more ghost pericytes (GP), and proliferative RECs. However, these retinal vasculopathies were alleviated in the DR+L-HY group and were improved in the DR+H-HY and DR+CD groups, as shown in **Figure 6B**. These preliminary results showed the improvement of hyperoside on diabetic retinal vasculopathy.

### 3.9 Comparisons of RVQ and E/P to evaluate the degree of retinal vasculopathy

As mentioned above, the RVQ and E/P were further calculated to quantitatively assess the alleviation of hyperoside in diabetic retinal vascular lesions. As shown in **Table 1**, the RVQ and E/P in the DR groups were significantly higher than those in NC [30.33 ± 3.83 *vs*. 14.83 ± 2.04 (*p* < 0.01) and 2.43 ± 0.22 *vs*. 1.03 ± 0.12 (*p* < 0.01)]. However, all RVQ and E/P in the DR+L-HY, DR+H-HY, and DR+CD groups were significantly lower than those in DR. In addition, the RVQ and E/P in DR+H-HY were significantly lower than those in DR+L-HY [19.83 ± 2.32 *vs*. 24.50 ± 2.88 (*p* < 0.05) and 1.52 ± 0.18 *vs*. 1.83 ± 0.13 (*p* < 0.05)]. These results indicate that hyperoside could alleviate the severity of retinal vasculopathy in DR rats and that the alleviation effect was more strengthened with the increase of hyperoside dose.

**Table 1:** Comparison of the RVQ and E/P of the retinal capillaries in different groups.

	NC (*n* = 6)	DR (*n* = 6)	DR+L-HY (*n* = 6)	DR+H-HY (*n* = 6)	DR+CD (*n* = 7)
RVQ	14.83 ± 2.04	30.33 ± 3.83**	24.50 ± 2.88^##^	19.83 ± 2.32^##,Δ^	19.86 ± 1.57^##^
E/P	1.03 ± 0.12	2.43 ± 0.22**	1.83 ± 0.13^##^	1.52 ± 0.18^##,Δ^	1.27 ± 0.07^##^

Data are presented as the mean ± standard deviation. A *p* < 0.05 is statistically significant.

*RVQ*, retinal vascular quantity; *E/P*, endothelial cell-to-pericyte ratio; *NC*, normal control; *DR*, diabetic retinopathy; *L-HY*, low-dose hyperoside; *H-HY*, high-dose hyperoside; *CD*, calcium dobesilate.

**p < 0.01 *vs*. NC; ^##^ *p* < 0.01 *vs*. DR; ^Δ^ *p* < 0.05 *vs*. DR+L-HY.

### 3.10 Hyperoside regulated the TGF-β1/miR-200b/VEGF expression in retinal tissues of DR rats

**Figure 7:**
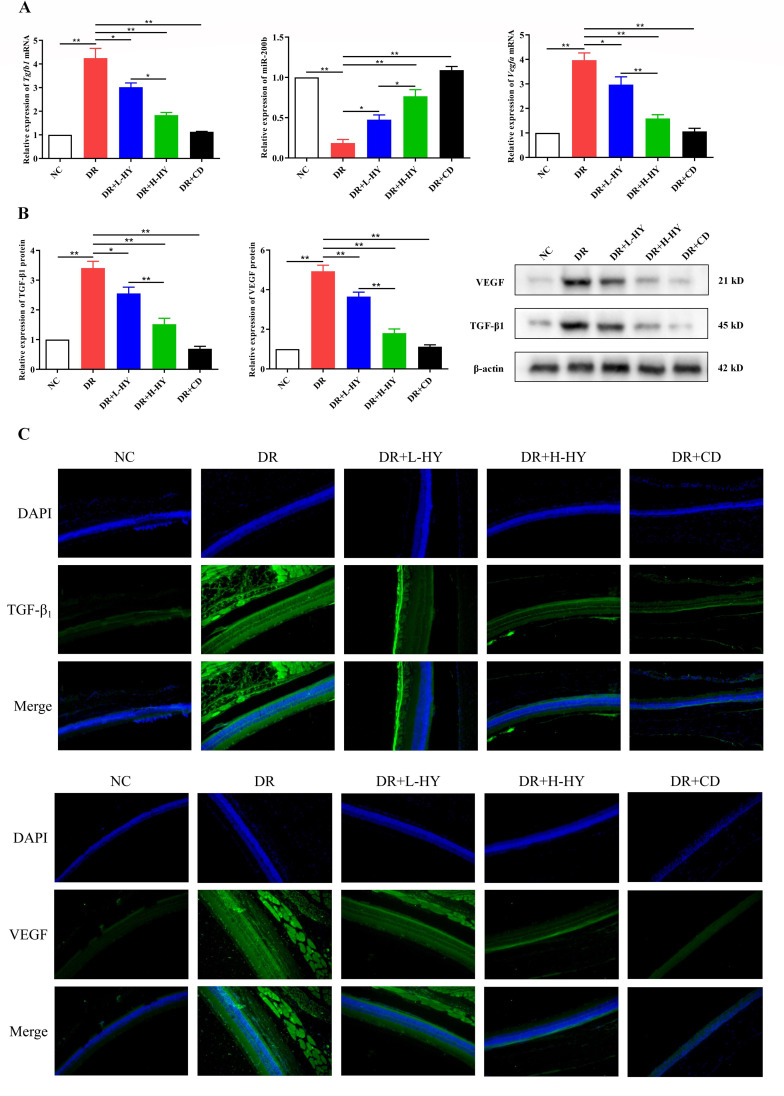
Hyperoside downregulated TGF-β1 and VEGF and upregulated miR-200b in the retinal tissues of diabetic retinopathy (DR) rats. **(A)** Expression of the *Tgfb1* and *Vegfa* mRNA and miR-200b in different groups assessed with quantitative reverse transcription PCR (qRT-PCR). **(B)** Expression of the TGF-β1 and VEGF proteins in different groups detected using Western blotting. **(C)** Representative images of the TGF-β1 and VEGF proteins detected using immunofluorescence (IF) in different groups (×200). *Green fluorescence* represents the fluorescein isothiocyanate (FITC)-labeled TGF-β1 and VEGF levels in retinal tissues, while *blue fluorescence* represents the DAPI-stained retinal cells. Both the PCR and Western blotting tests were repeated three times for statistical analysis. Data are presented as the mean ± standard deviation. A *p* < 0.05 is statistically significant. *p < 0.05, **p < 0.01.

Finally, the expressions of *Tgfb1*(TGF-β1), miR-200b, and *Vegfa*(VEGF) in the retinal tissues of all groups were compared using qRT-PCR, Western blotting, and immunofluorescence (IF). The mRNA and protein expressions of *Tgfb1* and *Vegfa* in DR were significantly higher than those in NC [4.25 ± 0.72 *vs*. 1.00 ± 0.00 (*p* < 0.01) and 3.41 ± 0.39 *vs*. 1.00 ± 0.00 (*p* < 0.01); 3.97 ± 0.51 *vs*. 1.00 ± 0.00 (*p* < 0.01) and 4.93 ± 0.53 *vs*. 1.00 ± 0.00 (*p* < 0.01), respectively] (**Figures 7A, B**), while the miR-200b in DR was significantly lower than that in NC (0.19 ± 0.07 *vs*. 1.00 ± 0.00, *p* < 0.01) (**Figure 7A**). However, hyperoside reversed the expressions of *Tgfb1*/miR-200b/*Vegfa* in the DR group. As shown in **Figures 7A, B**, both the low-dose (DR+L-HY) and high-dose hyperoside (DR+H-HY) and calcium dobesilate (DR+CD) all significantly inhibited the mRNA and protein levels of *Tgfb1* and *Vegfa* and upregulated the miR-200b levels compared with the DR groups [0.48 ± 0.10 *vs*. 0.19 ± 0.07 (*p* < 0.05), 0.77 ± 0.14 *vs*. 0.19 ± 0.07 (*p* < 0.01), and 1.09 ± 0.08 *vs*. 0.19 ± 0.07 (*p* < 0.01), respectively] (**Figure 7A**). The comparison between DR+L-HY and DR+H-HY further showed that the stronger regulation of *Tgfb1*, *Vegfa*, and miR-200b occurred along with the increase of hyperoside dose. Similar differences in TGF-β1 and VEGF in all groups are also shown in **Figure 7C**. In the DR group, the green fluorescence intensities representing the expression levels of TGF-β1 and VEGF were significantly stronger than those in the NC group. However, the green fluorescence intensity of the DR+L-HY group was significantly weaker than that of the DR group. In the DR+H-HY and DR+CD groups, the green fluorescence intensities were further weakened. Therefore, these data suggest that hyperoside could downregulate *Tgfb1* and *Vegfa*, but upregulate miR-200b, in the retinal tissues of DR rats.

## 4 Discussion

TGF-β is a protein family with multifunctional and bidirectional intercellular signaling. Different isoforms (e.g., TGF-β1, TGF-β2, and TGF-β3) are highly homologous in structure, and their functions are quite similar. TGF-βs are synthesized and secreted as precursor proteins, which are combined with the latency-associated peptide in an inactivated manner. After cleaving of the latency-associated peptide, the activated TGF-βs can bind to specific receptors on the cell surface and play essential roles in the growth, development, inflammation, repair, and host immunity of cells (9). Recent studies have shown that TGF-β1 is upregulated by HG and is considered a pro-inflammatory cytokine that could be implicated in the pathogenesis of DR (10). Elevated levels of TGF-β1 specifically bound to its receptor on the cell surface and transmitted the signal to intracellular Smad proteins (11), which continued to transmit the signal into the nucleus to inhibit the activity of the miR-200b promoter and reduce the expression of miR-200b (12, 13). miR-200b is an endogenous non-coding RNA that does not participate in protein coding, but is precisely bound to the 3′ non-coding region of the *VEGFA* mRNA to inhibit its translation and protein synthesis (14, 15). Previous studies have provided proof that the overexpression of VEGF plays an essential role in the dysfunction of RECs induced by HG and contributes to the destruction of the BRB in several blinding eye diseases, including DR (16–18). However, the detailed mechanisms have not been clearly illustrated. Therefore, evaluation of the TGF-β1/miR-200b/VEGF pathway in the pathogenesis of DR was the first aim of our study.

The CCK-8 assay was used to evaluate the proliferation of RECs. There are several methods for the determination of cell proliferation by detecting the replication activity of DNA. CCK-8 indirectly reflects the number of living cells by detecting the cell metabolic activity. However, it has advantages, such as its simple operation, high sensitivity, accurate results, good repeatability, low cytotoxicity, and the absence of radioactivity, among others. Both the DNA- (EdU, PCNA, and Ki-67) and metabolism-related tests (CCK-8) support the same conclusion uniformly. In this study, CCK-8 was used to confirm that the excessive proliferation of RECs was induced by HG, which has been applied in many studies previously (19, 20). The focus of this study was to confirm the proliferation of RECs induced by HG and inhibited by hyperoside. In addition, the migration and the tube formation of RECs were further assayed to evaluate the role of HG and hyperoside on the dysfunction of RECs. All of the results including those of CCK-8, Transwell, and tube formation of RECs should be sufficient to draw conclusions.

The results of our experiments *in vitro* showed that the proliferation, migration, and tube formation of RECs were more significantly related to the increase of glucose concentration, while this promotion effect was not observed in the same concentration of mannitol. These results suggest that the overproliferation of RECs and retinal angiogenesis were mainly driven by HG rather than high osmotic pressure. Similarly, HG remarkably induced an increase in the expression of *TGFB1* and *VEGFA*, but decreased the expression of miR-200b in RECs, consistent with the results of previous studies (21). However, after transfection of the miR-200b mimic into RECs under the HG condition, the mRNA level of *TGFB1* was also significantly elevated. The viability of RECs and the expression of *VEGFA* were noticeably decreased, while the expression of miR-200b was significantly increased. In contrast, after transfection of the miR-200b inhibitor into RECs under the NG condition, the viability of RECs and the expression of the *VEGFA* mRNA were significantly enhanced, while the expression of miR-200b was significantly reduced. The results of the above transfection experiments suggest that the activation of the TGF-β1/miR-200b/VEGF pathway played a pivotal role in the REC proliferation and retinal angiogenesis induced by HG. There are several mechanisms that result in DR, including inflammation, pyroptosis, retinal neovascularization, epigenetic modification, perturbation of the redox system, and the interference of miRNA. The TGF-β1/miR-200b/VEGF pathway may not be the only mechanism, but it must be a crucial mechanism in the pathogenesis of DR.

Our previous study provided proof that *A. manihot* could improve DR severity, the ETDRS vision score, macular edema, and the serum VEGF levels in type 2 diabetes (6). Hyperoside is a primary active ingredient of *A. manihot* (22), and the hyperoside content in *A. manihot* is notably higher compared with other herbal medicines. Hyperoside is also one of the flavonoid glycosides with anti-inflammatory, antioxidant, antidepressant, and anticancer effects (23). Recent studies have confirmed that hyperoside could improve the oxidative stress and inflammation induced by HG (24) and inhibit the overexpression of TGF-β1 under a HG condition. Can this explain the therapeutic mechanism of *A. manihot* against DR? What is the role of the TGF-β1/miR-200b/VEGF pathway in the treatment of DR with hyperoside? These issues need more illustration. In this study, further *in vitro* experiments have shown that both low- and high-concentration hyperoside could inhibit the proliferation, migration, and tube formation of RECs induced by HG, and this inhibition was obviously strengthened with the increase of hyperoside concentration. Hyperoside also significantly downregulated the activation of the TGF-β1/miR-200b/VEGF pathway in the REC proliferation induced by HG, and this regulation was noticeably enhanced with the increase of hyperoside concentration.

Furthermore, the efficacy of hyperoside on retinal damage in diabetic rats was assessed. During the retinal tissue procurement protocol for this experiment, a “post-fixation separation” approach was implemented for rat eyeballs prior to retinal isolation. Our rationale for adopting “post-fixation separation” rather than “fresh tissue dissection” was based on the following considerations:

1. Rat retinas connect with the retinal pigment epithelium (RPE) primarily through sparse microvilli without firm physical adhesion. Post-fixation separation benefits from a weakened retinal cup adhesion due to protein cross-linking reduction.
2. Fixation preserves the retinal laminar architecture integrity (e.g., ONL and the photoreceptor layer), minimizing the mechanical traction artifacts observed in fresh tissue dissection. Comparative experiments have demonstrated a 30% reduction in the photoreceptor outer segment fracture rates in post-fixation specimens.
3. Fixed tissue facilitates standardized processing for dehydration, embedding, and sectioning, which is particularly suitable for paraffin-embedded protocols (e.g., serial 4-μm sections for H&E staining).
4. While the traditional “whole cup sectioning” may better serve choroid/RPE studies (25), our focus on retinal laminar pathology (e.g., INL thickness and ganglion cell density) necessitates retinal isolation to eliminate choroidal vascular/pigment interference.

The results of the *in vivo* experiments showed that the retinal GCLs of the DR group were disordered and that the INL and ONL were sparsely arranged. It was suggested that the retinal damage in the DR group was relatively severe, and the lesion had broken through the BRB and spread to optic nerve cells. Furthermore, the use of E/P and RVQ as representative indicators to assess the degree of retinal capillary degeneration and neovascularization in DR was convincing (26). The results showed that the E/P and RVQ in the DR group were significantly higher than those in the NC group, while the REC proliferation and angiogenesis also noticeably increased in the DR group. In contrast, hyperoside could significantly improve the retinal tissue injury and vascular lesions in the DR group, and its efficacy was amplified with the elevation of its dose. These results preliminarily confirmed that hyperoside could treat DR.

The Western blotting, IF, and PCR results of this study showed that hyperoside also conspicuously decreased the expression of *Tgfb1* and *Vegfa* in the retinal tissues of DR rats, but markedly increased the expression of miR-200b. These results further confirmed the inhibitory effect of hyperoside on the TGF-β1/miR-200b/VEGF pathway. It should be noted that β-actin was selected as the loading control in this study. Although the close molecular weights of TGF-β1 (45 kDa) and β-actin (42 kDa) theoretically increase the risk of a band overlap during electrophoresis, previous studies have successfully validated β-actin as a reliable loading control for TGF-β1 detection under optimized electrophoretic conditions (27, 28). In our experiments, the 10% SDS-PAGE protocol with an extended run time (120 min at 100 V) demonstrated effective separation resolution. Furthermore, the expression trends of TGF-β1 in Western blotting aligned with the qRT-PCR data (mRNA level) **Figures 3** and **4**) and the IF results (**Figure 7**), indirectly corroborating the biological validity of our findings.

Comparison of the FBG levels of the DR rats in all groups at 0 w, 8 w, and 16 w showed that all of the FBG levels in the DR, DR+L-HY, DR+H-HY, and DR+CD groups were higher than those for the NC group, but no significant differences were found in all DR rat groups. These data suggest that the therapeutic effect of hyperoside on DR rats is not dependent on the decrease of blood glucose, which was different from previous studies reporting that some herbal medicines could improve diabetic complication with the hypoglycemic method (29, 30). However, it is known that the “metabolic memory” effect caused by long-term hyperglycemia can lead to an embarrassing situation that many DM patients cannot prevent the progression of diabetic complications despite significant improvement in blood glucose (31, 32). Interestingly, the positive control drug (calcium dobesilate) used in our *in vivo* experiment did not alleviate DR by lowering blood glucose levels either. Calcium dobesilate, a well-established agent for diabetic microangiopathy, exerts therapeutic effects in DR through mechanisms including antioxidant activity, reduction of vascular permeability, and inhibition of platelet aggregation (33, 34). It was selected as the positive control due to its documented efficacy in previous DR models (35), providing a robust benchmark for comparative analysis. Therefore, it is particularly important to explore effective methods to improve DR independent of glycemic control. The results of this study showed that hyperoside treated DR by inhibiting the TGF-β1/miR-200b/VEGF pathway rather than lowering the blood glucose. Hyperoside can be regarded as a potential effective complementary and alternative treatment for DR.

However, we also found inconsistencies in our findings with those of other studies. For example, Wu et al. (24) proposed that HG caused the apoptosis of RECs in rats, while hyperoside significantly inhibited the HG-induced apoptosis and promoted the proliferation of RECs. This is a highly intriguing phenomenon. Both our investigation and previous studies (36, 37) demonstrated that HG induced the excessive proliferation of RECs, which contradicted the findings reported in Wu et al. Upon meticulous analysis of the experimental methodology in Wu et al., it was identified that the RECs in their study were obtained through collagenase digestion of the rat retinal tissues, followed by filtration-based extraction. Given that RECs and RPCs are structurally adjacent components constituting both the BRB and retinal architecture, the direct extraction of RECs from intact rat ocular globes and retinal tissues for experimentation raises methodological concerns. Notably, the study by Wu et al. provided no explicit validation of the purity of RECs (e.g., via endothelial-specific markers such as CD31 or GLUT1). Coincidentally, a previous study (38) established that hyperglycemic conditions significantly enhanced apoptosis in RPCs. Therefore, if the REC preparations in Wu et al. contained RPC contaminants and our study utilized commercially sourced RECs (ACBRI-181, Cell Systems) with a rigorously validated cellular purity, this cross-contamination hypothesis could partially account for the discrepancies between their findings and our experimental outcomes.

Due to time and fund limitations, certain experiments such as the detection of hyperoside concentrations in the retina, the evaluation of REC proliferation with EdU or Ki-67, and tests of RPC loss inhibition by hyperoside were not performed. A pharmacokinetic study is indeed an important consideration in translational research, and RPC loss is also regarded as an essential pathogenic process in BRB dysfunction (39). Furthermore, appropriately increasing the sample size and experimental replicates would further strengthen the confidence intervals associated with the research data and conclusions. The completion of all related experiments will provide a more evidence-based explanation of the pathogenesis of DR and the pharmacological mechanisms of drug activity. These deficiencies will be improved in further studies.

## 5 Conclusions

In summary, the present study offers convincing evidence that hyperoside can improve the REC overproliferation and angiogenesis induced by HG and alleviate the retinal tissue injury and vascular lesions in DR rats. The therapeutic mechanism is possibly attributed to the regulation of the TGF-β1/miR-200b/VEGF pathway by hyperoside. These results can be taken as a novel exploration of treatment strategies for DR.

## Data Availability

The original contributions presented in the study are included in the article/supplementary material. Further inquiries can be directed to the corresponding author.
